# The Oxidative Stress Responsive Transcription Factor Pap1 Confers DNA Damage Resistance on Checkpoint-Deficient Fission Yeast Cells

**DOI:** 10.1371/journal.pone.0089936

**Published:** 2014-02-25

**Authors:** Carrie Belfield, Craig Queenan, Hui Rao, Kenji Kitamura, Nancy C. Walworth

**Affiliations:** 1 Department of Pharmacology, Rutgers Robert Wood Johnson Medical School, Piscataway, New Jersey, United States of America; 2 Graduate Program in Cellular and Molecular Pharmacology, Rutgers Graduate School of Biomedical Sciences, Piscataway, New Jersey, United States of America; 3 Center for Gene Science, Hiroshima University, Higashi-Hiroshima, Japan; 4 Member, Rutgers Cancer Institute of New Jersey, New Brunswick, New Jersey, United States of America; University of Minnesota, United States of America

## Abstract

Eukaryotic cells invoke mechanisms to promote survival when confronted with cellular stress or damage to the genome. The protein kinase Chk1 is an integral and conserved component of the DNA damage response pathway. Mutation or inhibition of Chk1 results in mitotic death when cells are exposed to DNA damage. Oxidative stress activates a pathway that results in nuclear accumulation of the bZIP transcription factor Pap1. We report the novel finding that fission yeast Pap1 confers resistance to drug- and non-drug-induced DNA damage even when the DNA damage checkpoint is compromised. Multi-copy expression of Pap1 restores growth to *chk1*-deficient cells exposed to camptothecin or hydroxyurea. Unexpectedly, increased Pap1 expression also promotes survival of *chk1*-deficient cells with mutations in genes encoding DNA ligase (*cdc17*) or DNA polymerase δ (*cdc6*), but not DNA replication initiation mutants. The ability of Pap1 to confer resistance to DNA damage was not specific to *chk1* mutants, as it also improved survival of *rad1*- and *rad9*-deficient cells in the presence of CPT. To confer resistance to DNA damage Pap1 must localize to the nucleus and be transcriptionally active.

## Introduction

Eukaryotic cells have distinct pathways that promote cell survival in the presence of DNA damage or other types of stress. In the fission yeast, *Schizosaccharomyces pombe*, a group of well conserved proteins makes up the DNA damage checkpoint pathway to prevent premature entry of cells from G2 to Mitosis during the cell cycle [1], [2]. The protein kinase Chk1, extensively studied in multiple systems as an essential component and transducer in this pathway, was first identified in the fission yeast [3], [4]. In the event of DNA damage, Chk1 is phosphorylated and its kinase activity increased, which requires the function of multiple Rad proteins including Rad3, the homologue of mammalian ATR [5]–[7]. Once activated, Chk1 can elicit a delay in mitotic entry by phosphorylating the Cdc25 phosphatase thereby maintaining it in an inactive state [8], [9]. The Cdc2 inhibitory protein kinase Wee1 is also critical to maintain Cdc2 tyrosine phosphorylation [10], [11]. The persistence of tyrosine phosphorylated Cdc2 serves to block cells in G2 allowing time to repair damaged DNA prior to progressing into mitosis [12].

*S. pombe* cells utilize additional signaling pathways to cope with other types of cellular stress. In the presence of oxidative stress induced by reactive oxygen species (ROS) or other oxidants from the environment, cells induce the expression of proteins including ROS scavengers and proteins to counteract the oxidative burst [13]–[17]. Expression of the genes encoding these proteins is mediated by the AP-1-like transcription factor Pap1 in fission yeast, and Yap1, in budding yeast [18], [19]. In unstressed cells, Pap1 shuttles transiently in and out of the nucleus, but appears predominantly cytoplasmic when visualized by light microscopy. However, in the presence of oxidative stress, Pap1 accumulates in the nucleus due to regulated nuclear export [18], [20]. Thus, changes in the intracellular redox state determine the cellular localization of Pap1.

Two cysteine-rich domains (CRDs) contribute to regulation of Pap1 localization [20]. The n-CRD is located at the center of the protein while the c-CRD is located at the C-terminus. The latter overlaps a nuclear export sequence (NES) in which one cysteine residue is embedded [21]. In the presence of oxidative stress, a disulfide bond forms between a cysteine residue in the n-CRD and one in the c-CRD, disrupting the function of the NES. This blocks access of the NES to the exportin Crm1 thereby preventing nuclear export of Pap1 until the redox state of the cell returns to normal [20]. Pap1 nuclear accumulation can also be induced by treatment of cells with diethylmaleate, which depletes glutathione [20] or the glycolytic metabolite methylglyoxal, which is reactive with lysine, arginine and cysteine residues [22].

The MAP kinase Sty1 indirectly regulates nuclear localization of Pap1 through an additional stress responsive transcription factor Atf1 [23]. In the presence of low levels of oxidative stress (<0.2 mM H_2_O_2_) Pap1 accumulates in the nucleus within 5 minutes, a response that is independent of Sty1. However, at higher levels of oxidative stress, (>1 mM H_2_O_2_), Pap1 nuclear accumulation is dependent on Sty1 and does not occur immediately, but rather is delayed for 30 to 45 minutes [24]. Thus, low levels of H_2_O_2_ directly lead to Pap1 nuclear localization, while at higher levels Pap1 is initially unresponsive [25]. In the presence of high concentrations of H_2_O_2_, formation of a cysteine sulfinic acid in the peroxiredoxin Tpx1 renders it inactive, which, in turn, prevents activation of Pap1 [14], [26]. Conversion of Tpx1 to an active form requires expression of the sulfiredoxin Srx1, which is a transcriptional target of Atf1 the transcription factor responsive to the Sty1 pathway [26]. Thus, restoration of the redox relay to Pap1 at higher concentrations of H_2_O_2_ requires Sty1 and Srx1 to mediate the recycling of Tpx1, thereby allowing nuclear retention of Pap1 [14].

In addition to its role in mediating resistance to oxidative stress, elevated expression of Pap1 confers resistance to various drugs, including caffeine, staurosporine and actinomycin D [27]–[31]. Here we report a novel role for Pap1 in conferring resistance to drugs that damage DNA as well as to drug-independent DNA damage caused by inactivation of proteins required for DNA replication. Pap1 improves survival of cells under these conditions even when the Chk1-dependent DNA damage checkpoint pathway is compromised.

## Materials and Methods

### Yeast Strains, Media, and Growth Conditions

Yeast strains used are listed in Table S1. All strains carrying plasmids were grown in pombe minimal medium made from Edinburgh Minimal Medium supplemented with adenine at 150 mg/L (PMA) and plated on yeast extract medium supplemented with adenine at 150 mg/L (YEA) [32]. Strains with integrated *pap1* under the control of the *nmt41* promoter were grown in liquid PMA medium and plated on PMA agar medium. Under these conditions expression from the *nmt41* promoter is derepressed. In some experiments, strains expressing *nmt41*-driven GFP-*pap1* were incubated in YEA media lacking added thiamine. While thiamine is assumed to be present in the media, sufficient GFP-*pap1* is expressed to visualize the protein by microscopy (see below) consistent with observations that the *nmt41* promoter is not completely off, even in the presence of thiamine [33]. Strains were typically grown at 30°C, unless a temperature-sensitive allele was present, in which case cells were grown at the permissive (25°C) and restrictive temperatures as indicated (see figures).

To screen for multi-copy suppressors of the CPT-sensitivity of a *chk1D469G* strain, cells (NW1509) were transformed with a genomic library, pTN-L1 [34], constructed in the *ars1*-based plasmid pAL-KS [35]. Approximately 30,000 transformants were replica-plated to YEA plates containing 10 µM CPT, and then replica-plated a second time from the CPT-containing plate to a second set of YEA plates with 10 µM CPT. Of 117 surviving colonies, 100 re-tested for growth on PMA plates. Following plasmid recovery, three of the original transformants contained plasmids with overlapping genomic fragments containing the *pap1* gene.

### Drug Treatment

Camptothecin lactone (CPT) was obtained from the Drug Synthesis and Chemistry Branch, Developmental Therapeutics Program, Division of Cancer Treatment, National Cancer Institute. Camptothecin and hydroxyurea (Sigma) were added to YEA and PMA plates at the concentrations indicated in the figures for use in spotting assays. For microscopy experiments, cells were treated with 40 µM CPT in liquid YEA medium for up to 4 hours. Hydrogen peroxide was added to YEA liquid cultures at the indicated concentration for 15 minutes.

### UV Sensitivity

One thousand cells were plated onto duplicate YEA plates and exposed to varying doses of UV light using a UV Stratalinker 2400 (Stratagene). Plates were incubated at 30°C until colonies formed. Colony counts were analyzed to determine the percentage of surviving cells relative to control plates containing an equal number of cells that were not exposed to UV light.

### Microscopy

To visualize GFP-tagged Pap1, cells were treated with hydrogen peroxide for 15 minutes followed by analysis using a Zeiss Axioplan fluorescence microscope. Cells were also treated for 15 minutes, then pelleted and resuspended in fresh medium to wash out the drug before analyzing a second time. To determine whether CPT treatment affects Pap1 localization, cells were incubated with 40 µM CPT at 30°C and samples were taken for visualization at varying time points up to 4 hours.

## Results

### Pap1 Induces Camptothecin Resistance in *chk1* Mutant Cells

**Figure 1:**
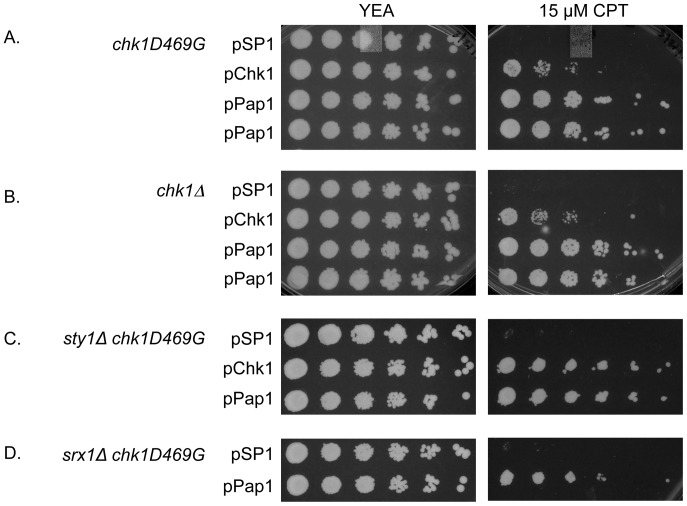
Pap1 rescues *chk1* mutant cells from DNA damage induced by CPT, independent of the Sty1 pathway. Five-fold serial dilutions of the indicated strains harboring either an empty vector (pSP1), wildtype *chk1* (pChk1), or wildtype *pap1* (pPap1) were plated on YEA agar medium in the absence or presence of 15 µM CPT and incubated at 30°C for 3–5 days. (A) *chk1D469G* (NW1509) (B) *chk1::ura4^+^* (NW158) (C) *sty1Δ chk1D469G* (NW2860) (D) *srx1Δ chk1D469G* (NW2861).

Previous studies identified a point mutation in *chk1* resulting in an amino acid substitution at position 469, D469G, in a conserved C-terminal domain that compromises Chk1 function [36]. Cells with the *chk1D469G* mutation are sensitive to DNA damaging agents including the topoisomerase I poison, camptothecin (CPT). In a screen for genes that confer multi-copy suppression of *chk1D469G*, we identified the transcription factor Pap1: when expressed from its native promoter on a multi-copy plasmid in *chk1D469G* mutant cells, Pap1 confers resistance to CPT (Figure 1A). Indeed, excess Pap1 (pPap1) confers more robust growth in the presence of CPT than can be achieved by restoring Chk1 function (pChk1) (Figure 1A). Pap1 also confers survival in the presence of CPT to cells with a deletion of *chk1*, indicating that Pap1 functions independently of Chk1 (Figure 1B).

### Pap1 Suppression of *chk1* Deficient Cells is not Dependent on Components of the Oxidative Stress Response Pathway

As a downstream effector of the oxidative stress response pathway, Pap1 is subject to regulation dependent on a redox mechanism mediated by the MAP kinase Sty1 under conditions of high oxidative stress [25]. We sought to determine whether Sty1 activation and peroxiredoxin recycling mediated by Srx1 are important for Pap1 rescue of *chk1*. We crossed both *sty1Δ* and *srx1Δ* (generously provided by Elena Hidalgo, Universitat Pompeu Fabra, Barcelona, Spain) mutants to *chk1D469G* and tested the ability of multi-copy Pap1 to restore growth in the presence of CPT. As shown in Figure 1C, we found that Pap1 was able to improve growth of the *chk1* mutant exposed to CPT independently of Sty1. In addition, deletion of *srx1* had no affect on the ability of Pap1 to restore growth in the presence of CPT (Figure 1D). These results suggest that Pap1 rescue of *chk1*-deficient cells exposed to CPT-induced DNA damage is independent of other oxidative stress response factors.

### Pap1 Rescues CPT Sensitivity of other DNA Damage Checkpoint Pathway Mutants

**Figure 2:**
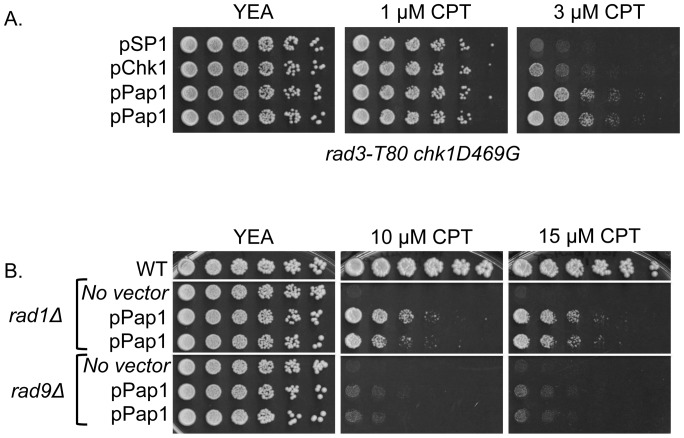
Pap1 rescues CPT sensitivity of other DNA damage checkpoint pathway mutants. (A) *rad3-T80 chk1D469G* strains carrying pSP1, pChk1, or pPap1 plasmids were spotted onto YEA medium in the absence or presence of 1 µM or 3 µM CPT. Plates were incubated at 30°C for 3–5 days. (B) *WT* cells, as well as *rad1Δ* and *rad9Δ* cells with or without pPap1 expression were spotted onto YEA medium in the absence or presence of 10 µM or 15 µM CPT.

In the DNA damage checkpoint pathway, the ATM-related protein kinase and homologue of mammalian ATR, Rad3, is responsible for Chk1 phosphorylation and activation [5], [6]. In order to determine whether Pap1 rescue of *chk1* relies on Rad3, we crossed a *rad3-T80* mutant to *chk1D469G* and expressed Pap1 on a multi-copy plasmid. Due to the greater sensitivity of the *rad3* mutant to CPT, serial dilutions of cells were plated on rich media containing 1 or 3 µM CPT. As shown in Figure 2A, Pap1 was able to restore growth in the presence of CPT even with *rad3* function compromised. The same results were observed in a *rad3-T80 chk1Δ* background (data not shown). These observations led us to consider whether Pap1 could rescue checkpoint mutants other than *chk1*, in particular, mutants deficient for either of two 9-1-1 complex components (Rad1 and Rad9) that function upstream of Chk1 in the DNA damage checkpoint pathway [6]. As shown in Figure 2B, serial dilution spotting of *rad1Δ* and *rad9Δ* cells expressing multiple copies of Pap1 showed that Pap1 robustly restores growth to *rad1Δ* cells and minimally, though perceptibly restores growth to *rad9Δ* cells in the presence of 10 or 15 µM CPT.

The DNA damage checkpoint pathway also contributes to cell survival when DNA replication is compromised by depletion of deoxyribonucleotides upon treatment with the ribonucleotide reductase inhibitor hydroxyurea (HU). While *chk1*-deficient cells show minimal sensitivity to HU, cells lacking upstream checkpoint pathway components *rad1*, *rad9* or *rad3* readily exhibit sensitivity to low concentrations of HU. As shown in Figure S1, multi-copy Pap1 is unable to suppress HU sensitivity of cells deficient for *rad3*, *rad1* or *rad9*.

### Pap1 Improves Survival of *chk1* Mutants Exposed to Non-Drug Induced DNA Damage

We further tested the ability of Pap1 to improve viability of *chk1* mutant cells exposed to DNA damage generated without the use of drugs. Temperature sensitive alleles of proteins important for DNA replication trigger Chk1 phosphorylation, indicative of checkpoint activation, and exhibit lethality at lower temperatures when Chk1 function is compromised [37], [38]. These observations suggest that abnormal DNA structures accumulated in such cells resemble DNA damage. Pap1 was introduced to cells with a null allele of *chk1* (*chk1::ura4^+^*) and selected replication mutants including *cdc17* (encoding the replicative DNA ligase), *cdc6* (encoding the lagging strand polymerase, DNA polymerase δ), *cdc21* (encoding Mcm4, a component of the MCM complex), and *cdc18* (encoding a loading factor for the MCM complex) [39]–[43]. While additional mutants were examined as well (e.g. *cdc24*, *cdc27* and *orp2*), only the *cdc6*, *cdc17*, *cdc18* and *cdc21* mutants reproducibly exhibited sufficiently clear *chk1*-dependent growth differences at permissive and restrictive temperature to be useful in assessing the ability of pPap1 to confer survival at restrictive temperature.

**Figure 3:**
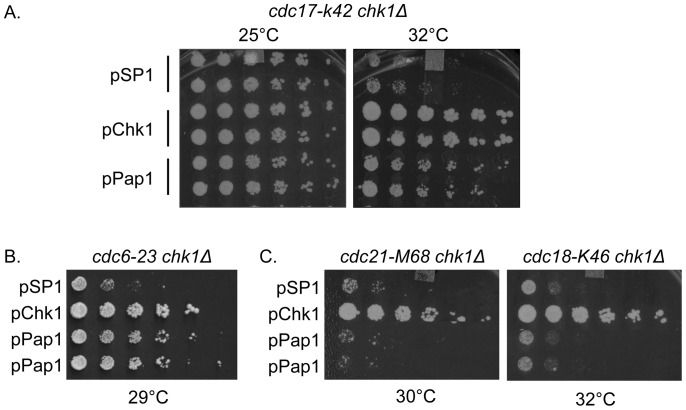
Pap1 improves survival of *chk1*-deficient cells with particular DNA replication defects. (A) A *chk1*-deficient strain with temperature sensitive DNA ligase, *cdc17-k42 chk1Δ* was transformed with an empty vector (pSP1), pChk1 or pPap1. Cells were grown overnight at 25°C, then serially diluted and spotted onto PMA plates at 25°C and the restrictive temperature for the double mutant of 32°C. (B) A *chk1*-deficient strain with temperature sensitive DNA polymerase δ, *cdc6–23 chk1Δ*, was transformed with an empty vector (pSP1), pChk1 or pPap1. Cells were grown overnight at 25°C, then serially diluted and spotted onto PMA plates at the restrictive temperature of 29°C. (C) *Chk1*-deficient strains with temperature sensitive alleles of an MCM subunit (*cdc21-M68 chk1Δ*) or the MCM complex loader (*cdc18-K46 chk1Δ*) were transformed with an empty vector (pSP1), pChk1 or pPap1. Cells were grown overnight at 25°C, then serially diluted and spotted onto PMA plates at temperatures restrictive for the double mutants, 30°C for *cdc21-M68 chk1Δ* and 32°C for *cdc18-K46 chk1Δ*.

The temperature sensitive DNA ligase mutant, *cdc17-K42*, is fully functional at 25°C, retains partial DNA ligase activity at 32°C and is non-functional at 36°C [39]. However, in combination with *chk1* mutations, double mutant cells become inviable at 32°C, a temperature that is tolerated when Chk1 is functional [44]. Therefore, we analyzed the ability of Pap1 to restore growth to *cdc17-K42 chk1Δ* cells at 32°C. As shown in Figure 3A, Pap1 clearly restores growth of *chk1Δ* cells in a DNA ligase-deficient background. Thus, elevated expression of Pap1 not only confers survival on checkpoint-deficient cells exposed to a DNA damaging drug, as shown in Figure 1, but also confers survival on checkpoint-defective cells that encounter unligated DNA by virtue of enzyme inactivation at elevated temperature.

As is the case for DNA ligase deficiency, the *chk1* dependence of survival at 29°C for the lagging strand DNA polymerase δ mutant (*cdc6–23*) is relieved by multi-copy expression of Pap1 (Figure 3B). In contrast, as shown in Figure 3C, Pap1 was not able to restore growth to DNA replication initiation mutants lacking *chk1* function (*cdc21-M68 chk1Δ* or *cdc18-K46 chk1Δ* double mutants at the restrictive temperatures for the double mutants of 30°C or 32°C, respectively). Since Pap1 also rescued checkpoint mutants exposed to CPT, we tested whether Pap1 could rescue *rad1* or *rad9* cells with compromised DNA ligase. In contrast to the data with CPT treatment and in keeping with that for HU, Pap1 is not able to suppress a DNA ligase mutant when combined with mutations in the 9-1-1 complex (Figure S2).

### Pap1 does not Prevent Mitotic Catastrophe or Delay the Cell Cycle

**Figure 4:**
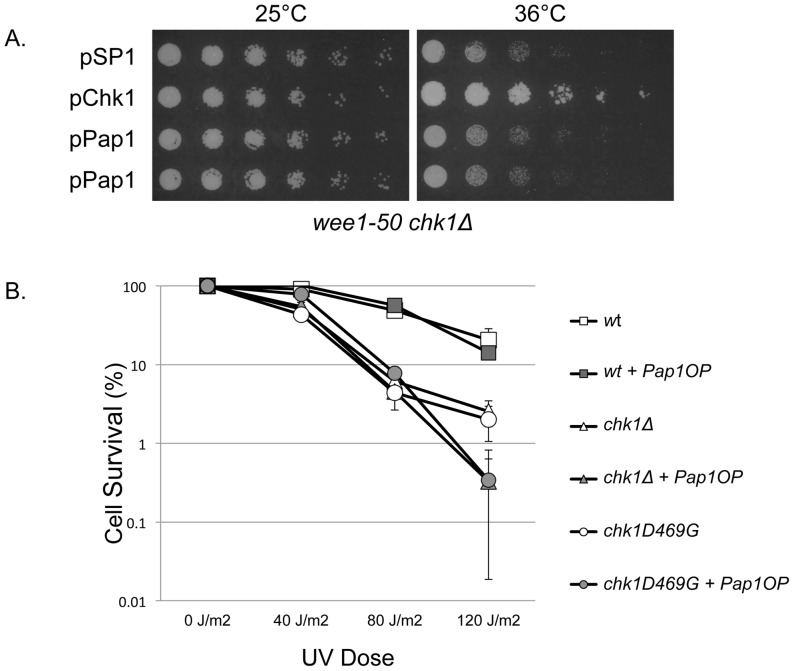
Pap1 does not rescue mitotic catastrophe due to loss of Cdc2 tyrosine 15 phosphorylation or UV sensitivity of *chk1*-deficient cells. (A) Transformants of a *wee1–50 chk1Δ* double mutant strain carrying pSP1, pChk1, or pPap1 were spotted onto YEA agar medium and incubated at the permissive temperature of 25°C or restrictive temperature of 36°C for 3–5 days. (B) *WT, chk1Δ, and chk1D469G* cells without or with Pap1 overexpression were plated on YEA agar medium and exposed to 0, 40, 80, and 120 J/m^2^ of UV light. Plates were incubated at 30°C for 3 to 5 days until colonies formed.

Fission yeast cells enter mitosis at a consistent cell size. Mutations that reduce or enhance tyrosine phosphorylation of the cyclin-dependent kinase Cdc2 promote or delay mitotic entry. To determine whether Pap1 rescues *chk1*-deficient cells simply by delaying cell cycle progression, we measured individual cell lengths of a population of cells expressing Pap1. The cell length distribution did not increase for cells expressing Pap1 as compared to cells with an empty vector (data not shown), as would be expected for cells experiencing a delay in mitotic entry. The protein kinase Wee1 plays a critical role in limiting activation of Cdc2 at the G2/M transition [10], [45], [46] and acts in opposition to the Cdc25 phosphatase that dephosphorylates Cdc2 thereby triggering mitotic entry [47], [48]. Deletion of *chk1* in the *wee1–50* background results in mitotic catastrophe wherein cells enter mitosis prior to completion of DNA replication leading to cell death at the restrictive temperature for *wee1–50* [3]. In order to determine if Pap1 is capable of restoring survival to cells subject to mitotic catastrophe, we transformed the multi-copy Pap1 plasmid into the temperature sensitive *wee1–50 chk1Δ* double mutant. At the restrictive temperature of 36°C, we observed that Pap1 was unable to restore growth, suggesting that Pap1 does not act to rescue cells lacking *chk1* simply by delaying cell cycle progression or impacting regulatory events that control the activation of Cdc2 (Figure 4A).

### Pap1 does not Promote Survival of Asynchronous *chk1* Mutants Exposed to UV Light

**Figure 5:**
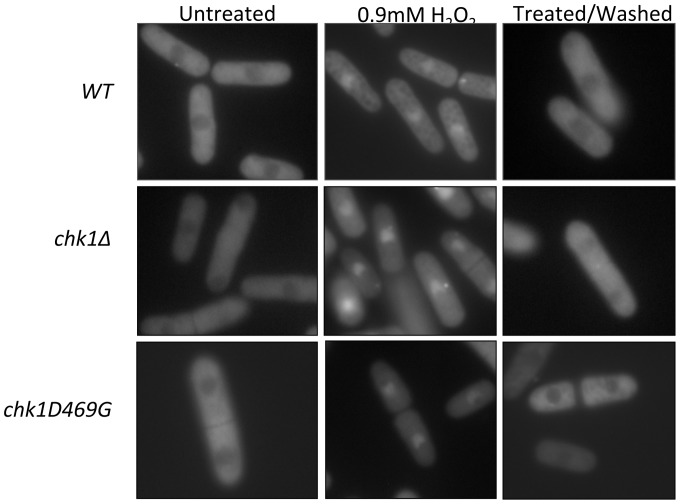
Chk1 does not affect Pap1 localization in response to H_2_O_2_. *WT, chk1Δ, and chk1D469G* strains expressing GFP-tagged Pap1 under control of the *nmt41* promoter were treated with 0.9 mM H_2_O_2_ for 15 minutes followed by analysis by fluorescence microscopy. Cells in the right column were treated with 0.9 mM H_2_O_2_ for 15 minutes and washed with fresh media to remove the drug before analysis.

Cells with mutations that abrogate the mitotic checkpoint exhibit sensitivity to DNA damage generated by exposure to ultraviolet light. To determine whether Pap1 affects survival of *chk1*-deficient cells exposed to DNA damage induced by UV light, we used a strain (a generous gift of Dr. Shelley Sazer, Baylor College of Medicine) with a chromosomally integrated allele of *pap1* tagged with GFP and expressed under control of the thiamine-repressible *nmt41* promoter [49], [50]. This strain was crossed to both *chk1Δ* and *chk1D469G* strains. Similar to suppression of CPT sensitivity with plasmid-expressed *pap1*, expression of GFP-*pap1* from the integrated *nmt41* promoter suppresses the CPT sensitivity of the *chk1D469G* and *chk1Δ* strains (data not shown). As shown in Figure 4B, when an asynchronous population of cells were exposed to DNA damage generated by UV light, we observed that expression of Pap1 had no effect on survival of cells with wild-type *chk1*, nor did Pap1 improve survival of *chk1*-deficient cells. Examination of these strains by fluorescence microscopy confirmed that GFP-Pap1 is expressed from the *nmt41* promoter when cells are grown in YEA (see Fig. 5). Thus, elevated Pap1 expression promotes survival of *chk1*-deficient cells exposed to DNA damage generated by CPT, HU, inactivation of DNA ligase or DNA polymerase δ, but does not improve survival when cells encounter DNA damage generated in asynchronous cells by UV light or when DNA replication initiation is compromised.

### Nuclear Accumulation of Pap1 in Cells Exposed to Oxidative Stress is Independent of Chk1 Function

In unstressed cells, Pap1 is found predominantly in the cytoplasm. However, in the presence of oxidative stress, such as exposure to H_2_O_2_, Pap1 briefly accumulates in the nucleus as the result of a block to nuclear export [18], [20]. An inverse relationship has been reported between the concentration of H_2_O_2_ and the time required to detect Pap1 nuclear accumulation [23]. In the presence of 0.2 mM H_2_O_2_, Pap1 accumulates in the nucleus after only 5 minutes of treatment and relocates to the cytoplasm within 15 minutes of exposure. In contrast, cells exposed to higher doses (1 or 5 mM) of H_2_O_2_ initially retain Pap1 in the cytoplasm after exposure, but accumulate Pap1 in the nucleus at later times, in a dose dependent manner, 30 and 70 minutes, respectively [23]. In order to determine if Chk1 has an effect on the normal localization of Pap1, we observed *chk1*-defective strains with GFP-tagged Pap1 integrated in the genome. All cells were grown to mid-log phase and treated with 0.9 mM H_2_O_2_ for 15 minutes before analysis. As expected, in untreated cells, Pap1 is detectable in the cytoplasm in wild-type, as well as *chk1* mutant cells. After treatment with H_2_O_2_, Pap1 accumulated in the nucleus regardless of *chk1* function (Figure 5). Thus, Chk1 is not required for the localization of Pap1 to the nucleus in response to oxidative stress.

### Pap1 Remains in the Cytoplasm in CPT-treated Cells

**Figure 6:**
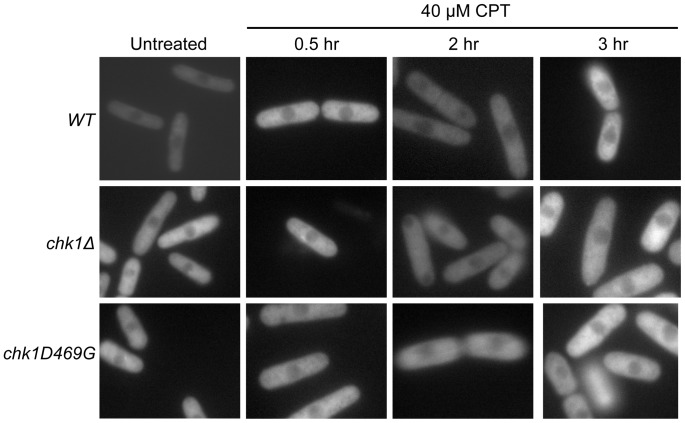
Pap1 does not appear to accumulate in the nucleus in response to CPT. *WT, chk1Δ, and chk1D469G* strains expressing GFP-tagged Pap1 under control of the *nmt41* promoter were exposed to 40 µM CPT at 30°C. Cells were collected after 30 minutes, 2 hours and 3 hours for analysis by fluorescence microscopy.

Since Pap1 compensates for lack of *chk1* function in cells exposed to DNA damage, we asked whether Pap1 localizes to the nucleus when cells are exposed to CPT. Exposure of fission yeast cells to 40 µM CPT induces a checkpoint response within 2 hours of treatment, accompanied by Chk1 phosphorylation, association of Chk1 with the 14-3-3 protein Rad24, and Chk1 nuclear localization [6], [51]–[53]. Indeed Chk1 phosphorylation is detected within 30 minutes of exposure to 2 µM CPT [53]. Using strains with *pap1* expression under control of the integrated *nmt41* promoter, we analyzed Pap1 localization at various times in CPT-treated cells. As shown in Figure 6, we were unable to detect nuclear localization of Pap1 after 0.5, 2, or 3 hours of CPT treatment, regardless of whether Chk1 is wild-type or mutant. Thus, despite its known role as a transcription factor, Pap1 appears cytoplasmic in cells that are restored to viability when Pap1 is overexpressed. These results led us to test whether nuclear localization of Pap1 is required for Pap1 to restore survival of cells exposed to different types of DNA damage.

### Transcriptionally Active Pap1 that Localizes to the Nucleus Improves Survival of *chk1* Mutant Cells in Response to Various Types of DNA Damage

**Figure 7:**
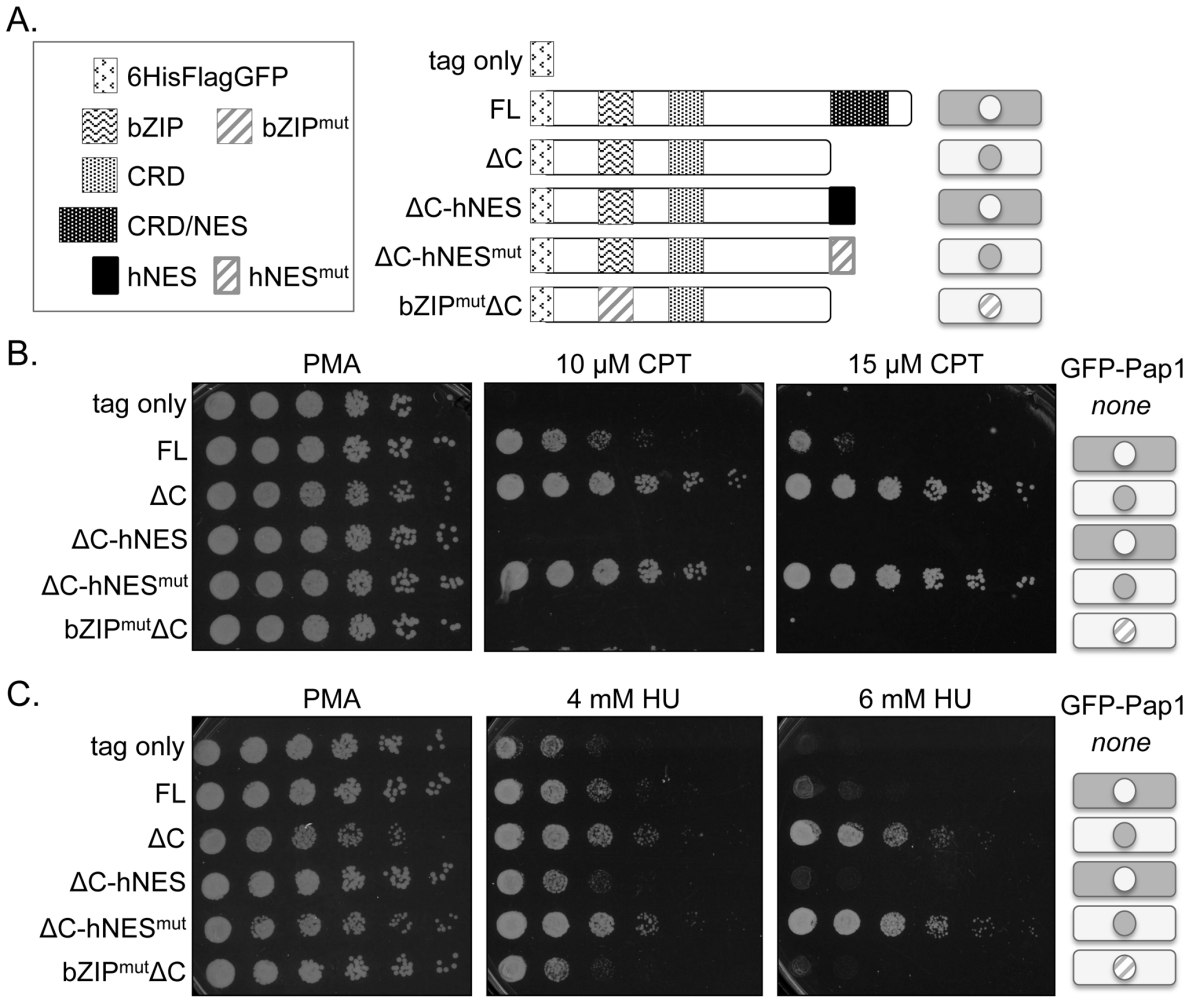
Pap1 must localize to the nucleus and be transcriptionally active to confer resistance to DNA damage. (A) Schematic of Pap1 domains and mutants harboring deletions. Localization of the mutant proteins as monitored by fluorescence microscopy is shown: shading in the cytoplasm represents cytoplasmic localization; shading in the nucleus represents nuclear localization; the hatched nucleus for the bZIPmutΔC mutant represents nuclear localization of a transcriptionally-defective protein. Localization data is reported in Kitamura et al. [54] and was confirmed for the indicated strains (data not shown). (B) Cells of a *chk1D469G* strain with integrated alleles of the indicated Pap1 constructs expressed under control of the *nmt41* promoter were assayed on PMA media for survival in the presence of 10 or 15 µM CPT. (C) Strains as in B were assayed on PMA media for survival in the presence of 4 or 6 mM HU.

In the absence of oxidative stress, Pap1 is predominantly localized to the cytoplasm. In cells treated with CPT, Pap1 appears cytoplasmic, yet *chk1*-deficient cells expressing Pap1 are restored to viability. The level of nuclear Pap1 necessary to promote survival of DNA damaged cells may be below that which is apparent by imaging GFP-tagged Pap1. Alternatively, Pap1 might function through a novel mechanism, perhaps independent of its transcriptional activity. To assess this possibility, we evaluated a set of Pap1 localization mutants for their ability to restore survival to *chk1*-deficient cells exposed to DNA damaging agents [54]. Each of the mutants, illustrated schematically in Figure 7A, encodes an integrated gene encoding a version of Pap1 with His, Flag and GFP tags (referred to as HFG) fused at the N-terminus of Pap1, with expression controlled by the *nmt41* promoter. The HFG tag was also integrated in the genome fused to full-length wild-type Pap1 (HFG-Pap1-FL) and without fusion to Pap1 as a negative control (tag only). Mutants affecting either localization or transcription activity of Pap1 were analyzed. As described previously [54], confirmed in our laboratory (data not shown) and represented schematically in Figure 7A, deletion of the C-terminal nuclear export sequence (NES) renders Pap1 constitutively nuclear (HFG-Pap1ΔC), while addition to Pap1ΔC of a heterologous NES from human protein kinase A renders Pap1 constitutively cytoplasmic (HFG-Pap1ΔC-hNES). Mutation of the heterologous NES also results in constitutive nuclear localization of Pap1 (HFG-Pap1ΔC-hNES^mut^). The combination of a point mutation in the bZIP domain that inactivates the transcriptional activity of Pap1, with deletion of the C-terminal NES (HFG-Pap1-bZIP^mut^ΔC), renders Pap1 constitutively nuclear, but non-functional as a transcription factor. Each of these Pap1 mutants was crossed to *chk1Δ* and *chk1D469G* strains and tested for the ability to improve survival of checkpoint-defective cells exposed to various forms of DNA damage.

As shown in Figure 7B, the constitutively nuclear, but transcriptionally inactive Pap1 mutant (HFG-Pap1-bZIP^mut^ΔC) does not restore growth to *chk1*-deficient cells exposed to CPT. This dependence on the bZIP domain supports the hypothesis that Pap1 functions as a transcription factor to rescue *chk1* mutant cells in response to DNA damage. Given this, we expected the constitutively nuclear Pap1 mutants to improve survival. Indeed, in the presence of CPT, the Pap1 mutants ΔC and ΔC-hNES^mut^, both of which are constitutively nuclear ([54] and data not shown), improve survival even more robustly than does full length Pap1 (FL). In contrast, the constitutively cytoplasmic Pap1, ΔC-NES, fails to improve survival, as though Pap1 were not overexpressed at all (compare to tag only control strain). In the presence of 4 or 6 mM hydroxyurea (HU), constitutively nuclear Pap1 also robustly restores growth (Figure 7C). When the Pap1 mutants were examined in a *chk1* null (*chk1Δ*) background and exposed to CPT or HU, results were identical to those shown for the *chk1D469G* mutant (Figure S3).

**Figure 8:**
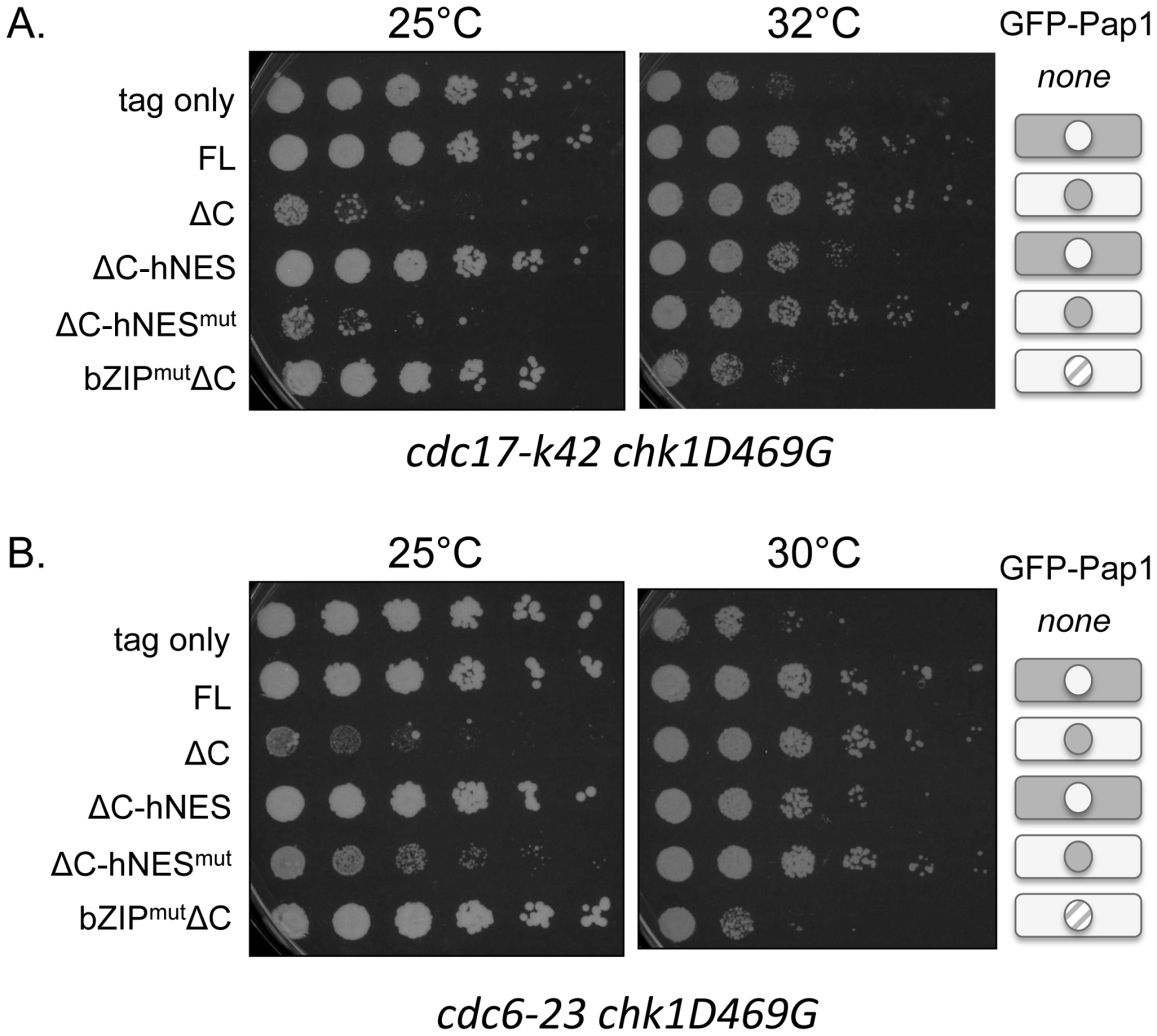
Nuclear Pap1 improves growth of checkpoint-deficient cells with defective DNA ligase or DNA polymerase δ. (A,B) Checkpoint and DNA ligase-deficient *cdc17-K42 chk1D469G* double mutants (A) or checkpoint and DNA polymerase δ-deficient *cdc6–23 chk1D469G* double mutants (B) expressing the Pap1 constructs described in figure 7A were spotted onto PMA medium and incubated at 25° and 32°C.

The Pap1 mutants were also crossed to the replication mutants *cdc17-K42* and *cdc6–23* in combination with *chk1D469G*. As shown in Figure 2A and 2B, these double mutant combinations exhibit synthetic growth defects at semi-permissive temperature for the *cdc* mutant, which can be suppressed by overexpression of Pap1. In both of these backgrounds, the FL, ΔC, and ΔC-hNES^mut^ constructs were able to restore growth at restrictive temperature, even while constitutively nuclear Pap1 (ΔC and ΔC-hNES^mut^) has a deleterious effect on growth at the nominally permissive temperature of 25°C (Figure 8). The constitutively cytoplasmic ΔC-hNES Pap1, confers a level of growth intermediate between that seen with the transcriptionally inactive Pap1 and nuclear Pap1. Collectively, these results indicate that transcriptionally active, nuclear Pap1 efficiently confers resistance to DNA damage, whether generated by drugs or inactivation of DNA replication proteins.

## Discussion

Pap1 has an established role as a transcription factor that protects cells exposed to oxidative stress [16]. Pap1 is also found, upon overexpression or deletion, to confer resistance to drugs including staurosporine, actinomycin, caffeine, brefeldin A and others [27]–[31]. Here, we report an additional role for Pap1 in conferring resistance to DNA damage generated either by drug treatment with camptothecin (CPT) or hydroxyurea (HU), or through the loss of function of proteins required for DNA replication. It has been postulated that Pap1 promotes drug resistance through upregulation of membrane-associated efflux pumps [18], [55]–[57]. While such a mechanism could explain the ability of elevated Pap1 to confer resistance to CPT or HU, it does not account for the ability of elevated Pap1 to confer resistance to DNA damage generated by mutations that compromise the activity of DNA ligase or DNA polymerase δ (Figure 3). Significantly, Pap1 confers DNA damage resistance on cells with mutations that compromise the DNA damage checkpoint pathway, which normally restrains mitotic entry when DNA damage or replication blocks are present [58].

Multicopy expression of Pap1 confers resistance to camptothecin (Figure 1) and to hydroxyurea (Figure 7). Camptothecin (CPT) poisons type I topoisomerases, thereby creating a block to DNA replication fork progression, which results in DNA double strand breaks [59], [60]. Hydroxyurea (HU) inhibits ribonucleotide reductase, which blocks DNA replication and can lead to replication fork collapse [61]. Interestingly, the ability of Pap1 to promote resistance to CPT is fully independent of *chk1* as elevated Pap1 can suppress the CPT sensitivity of a *chk1* deletion strain (Figure 1B). In addition, Pap1 promotes resistance to CPT in cells with mutations in the checkpoint proteins encoded by *rad1* and *rad9*, components of the PCNA-like clamp that loads to DNA damage sites, or *rad3*, which encodes the fission yeast homologue of the protein kinase ATR (Figure 2). While the ability of Pap1 to rescue the *rad1* and *rad3* mutants is robust, rescue of the *rad9* mutant is less so. Perhaps this observation reflects the unique role of Rad9 within the checkpoint-sliding clamp as the phosphoacceptor protein that interacts with Rad4^TopBP1^ to promote assembly of the checkpoint complex with Crb2^53BP1^ [62]–[64]. The absence of Rad9 protein would not only compromise the clamp, but would prevent any assembly of the Crb2 complex, which recruits and activates Chk1. As compared to requirement for bypassing loss of *rad1*, the ability to bypass the need for *rad9* would thus require substitution of additional functions.

While expression of Pap1 confers resistance to HU on cells deficient for *chk1* (Figure 7C), Pap1 does not confer resistance to HU on cells with mutations in *rad1*, *rad3* or *rad9* (Figure S1B). Thus, the ability of Pap1 to promote survival in the presence of HU is dependent on the 9-1-1 complex and Rad3, but is independent of Chk1, which functions downstream of both the 9-1-1 complex and Rad3 [6].

Like exposure to DNA damage, inactivation of specific DNA replication machinery components leads to checkpoint activation and a dependence on Chk1 for survival [3], [38]. While Pap1 improves survival of *chk1*-deficient cells with mutations either in DNA ligase (encoded by *cdc17*) or DNA polymerase δ (encoded by *cdc6*), Pap1 does not restore growth when replication initiation proteins Mcm4 (encoded by *cdc21*) or the MCM complex loader (encoded by *cdc18*) are compromised (Figure 3). The ability of Pap1 to restore growth to DNA replication mutants deficient for Chk1 is confined to a subset of replication mutants, which could reflect the severity of the defect conferred by the mutant, or some degree of specificity for the nature of the damage which does or does not permit Pap1 to restore growth. Notably, while Pap1 confers resistance to *chk1*-deficient cells with compromised DNA ligase, Pap1 does not confer resistance to *rad1-* or *rad9*-deficient cells with mutation of DNA ligase (Figure S2). It has been hypothesized that the 9-1-1 complex is recruited to Okazaki fragments on the lagging strand to initiate a checkpoint signal [65]. Perhaps Pap1 can rescue cells with a *chk1*-deficiency because 9-1-1 complex loading still occurs in the DNA ligase defective strain lacking Chk1. We can only speculate as to why this might be the case: perhaps the mechanism Pap1 uses to promote survival of checkpoint-deficient cells is influential only if a DNA damage recognition signal is established. When fork collapse is caused by HU treatment, Pap1 rescues *chk1*-deficient cells, but cannot rescue cells deficient for the 9-1-1 complex or the Rad3 kinase recruited by 9-1-1, leading us to suggest that a similar requirement for establishing the DNA damage signal in the presence of HU is necessary for Pap1 to promote survival. We note that Pap1 rescues to a variable degree the CPT sensitivity of mutants lacking any of the checkpoint components: Chk1, the 9-1-1 complex or Rad3. As the DNA double strand breaks CPT generates are detected by a set of damage recognition proteins distinct from the 9-1-1 complex, the 9-1-1 independence of Pap1 rescue is consistent. Presumably, the absence of *chk1*, which typically allows death in the absence of Pap1 overexpression, is far enough downstream of the damage signal to allow Pap1 overexpression to rescue cells so long as the damage signal is initiated. Curiously, mutations that affect initiation of DNA replication, including a mutation in one of the MCM complex subunits, or mutation of Cdc18, the protein that loads the MCM complex, are not subject to suppression caused by elevation of Pap1.

Chk1 phosphorylation in response to DNA damage results in a mobility shift on an SDS-PAGE gel detectable by Western blot [6]. When multiple copies of Pap1 are expressed in cells subsequently exposed to CPT or HU, Chk1 still becomes phosphorylated (data not shown). Since signaling to Chk1 remains intact when Pap1 expression is elevated, we presume that DNA damage is generated in *chk1*-deficient cells, but mitotic cell death, the typical consequence of that damage in checkpoint-deficient cells, does not occur. How cells with elevated levels of Pap1 tolerate the presence of damaged DNA, or bypass the need to impose a sustained block to mitosis remains to be determined. It is known that imposition of a G2 delay to mitosis by inactivation of the Cdc25 phosphatase responsible for activating the cyclin-dependent kinase Cdc2 can restore viability to *chk1*-deficient cells exposed to DNA damage [3]. If elevated expression of Pap1 were simply delaying the cell cycle thereby bypassing the need for Chk1, then elevated Pap1 should rescue *chk1*-deficient cells exposed to any type of DNA damage. However, Pap1 does not rescue *chk1*-deficient cells exposed to UV light (Figure 4) arguing against this possibility. As an AP-1 transcription factor, the consequences of Pap1 overexpression could be many and varied. Thus, determining the mechanism by which elevated Pap1 suppresses DNA damage sensitivity of *chk1*-deficient cells will require further investigation.

In response to oxidative stress, Pap1 is known to accumulate in the nucleus as a result of oxidation-dependent disulfide bond formation within the C-terminal region of the protein thereby altering accessibility of the nuclear export sequence. We did not observe nuclear accumulation of Pap1 in response to CPT (Figure 6), suggesting that even if CPT leads to the generation of ROS in *S. pombe*, the levels of oxidative stress are not sufficient to lead to nuclear accumulation of Pap1. There is evidence in the literature to suggest that exposure of mammalian or plant cells to CPT generates ROS [66]–[68]. However, at least one report suggests that CPT exposure may not generate significant levels of ROS in fission yeast [69]. The fission yeast Rad52 homologue is phosphorylated upon treatment with agents known to induce ROS, including UV-A and H_2_O_2_, while exposure to UV-C or 20 µM CPT does not lead to Rad52 phosphorylation [69]. Though indirect, this observation suggests that exposure of *S. pombe* cells to CPT does not result in robust generation of ROS and is likewise consistent with our observation that Pap1 does not accumulate in the nucleus in CPT-treated cells.

While nuclear accumulation of Pap1 is not detectable in cells exposed to CPT, nuclear localization of Pap1 is critical for its ability to promote survival in cells exposed to DNA damage (Figures 7 and 8). Studies with Pap1 mutants that localize predominantly to the nucleus or cytoplasm support the need for Pap1 to localize to the nucleus in order to promote survival of checkpoint-deficient cells in the presence of DNA damage. Likewise, the observation that the Pap1-bZIP mutant does not confer survival suggests that Pap1 must also be transcriptionally active. Whether Pap1 induces expression of specific genes to create a cellular context that allows survival in the absence of checkpoint function remains to be determined. Regardless of mechanism, it is important to appreciate that cell killing by agents that damage DNA, including clinically relevant topoisomerase I poisons, can be overcome by elevated expression of a stress responsive transcription factor that was previously unlinked to DNA damaging agents.

## Supporting Information

Figure S1 **Pap1 does not rescue HU sensitivity of DNA damage checkpoint pathway mutants.** (A) *rad3-T80 chk1D469G* strains carrying pSP1, pChk1, or pPap1 plasmids were spotted onto YEA medium in the absence or presence of 1.8 mM or 2 mM HU. Plates were incubated at 30°C for 3–5 days. (B) Wildtype cells, as well as *rad1Δ* and *rad9Δ* cells with or without pPap1 expression were spotted onto YEA medium in the absence or presence of 1.5 mM or 3 mM HU.
(TIF)

Figure S2 **Pap1 is unable to rescue 9-1-1 complex mutants with compromised DNA ligase.** Strains of the indicated genotypes were transformed with the indicated plasmids and grown to mid-log phase at 25°C. Serial dilutions were spotted at 25°C or restrictive temperature for the double mutants, as indicated, and incubated for 3 to 5 days. (A) *cdc17-K42 rad1-1* (B) *cdc17-K42 rad9Δ*.
(TIF)

Figure S3 **Pap1 that is nuclear and transcriptionally active confers resistance to cells deleted for** ***chk1*** **when exposed to CPT or HU.** (A) Cells of a *chk1::ura4* strain with integrated alleles of the indicated Pap1 constructs expressed under control of the *nmt41* promoter were assayed for survival in the presence of 10 or 15 µM CPT. (B) Strains as in A were assayed for survival in the presence of 4 or 6 mM HU.
(TIF)

Table S1 **Yeast Strains Used.**
(DOCX)
