# Supplementary figures and images for: The Oxidative Stress Responsive Transcription Factor Pap1 Confers DNA Damage Resistance on Checkpoint-Deficient Fission Yeast Cells
Source: PLoS One. 2014 Feb 25;9(2):e89936. doi: 10.1371/journal.pone.0089936 (PMC3934961; doi:10.1371/journal.pone.0089936)

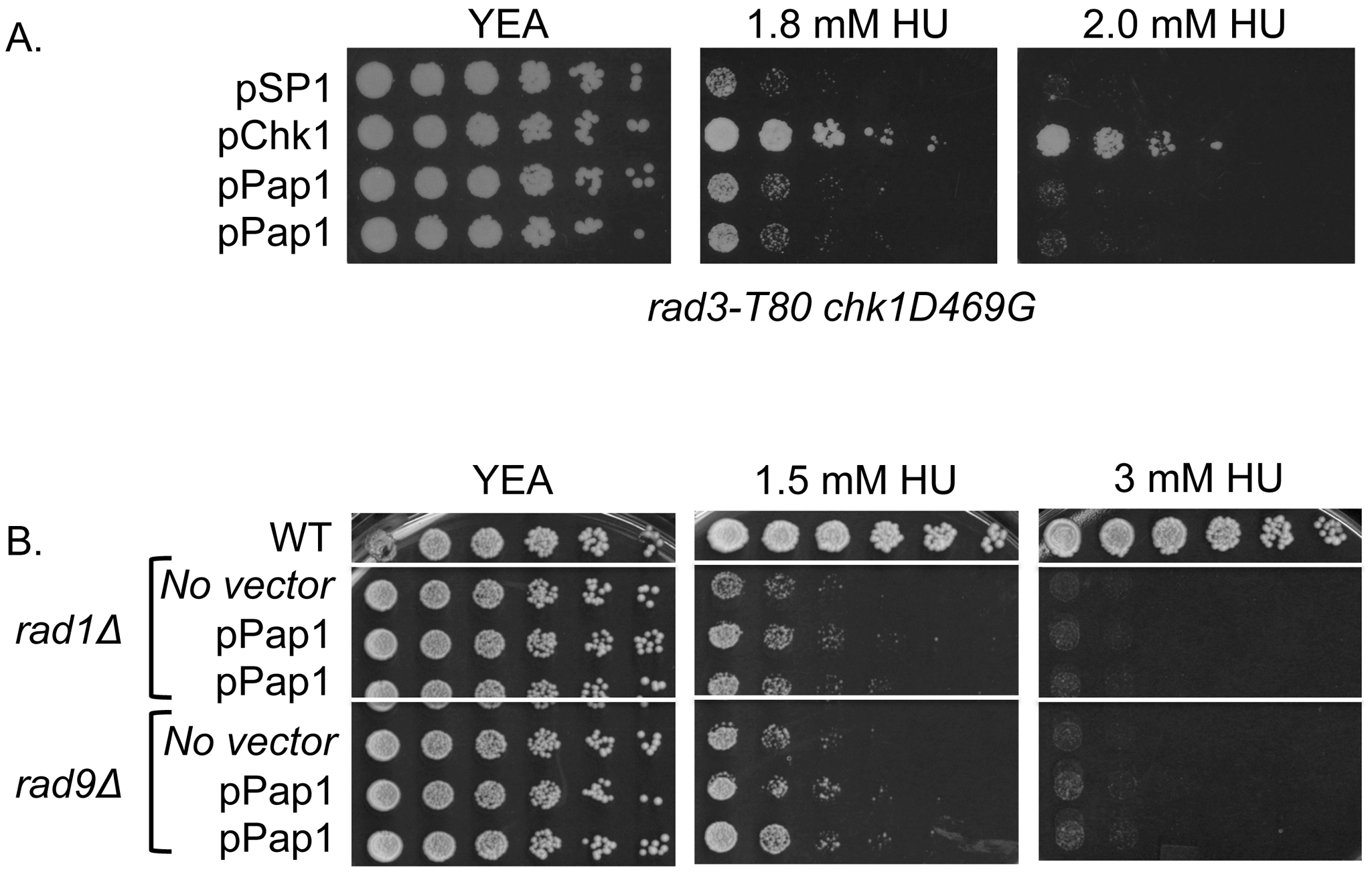

Supplement: Figure S1 — Pap1 does not rescue HU sensitivity of DNA damage checkpoint pathway mutants. (A) rad3-T80 chk1D469G strains carrying pSP1, pChk1, or pPap1 plasmids were spotted onto YEA medium in the absence or presence of 1.8 mM or 2 mM HU. Plates were incubated at 30°C for 3–5 days. (B) Wildtype cells, as well as rad1Δ and rad9Δ cells with or without pPap1 expression were spotted onto YEA medium in the absence or presence of 1.5 mM or 3 mM HU. (TIF) [file pone.0089936.s001.tif]

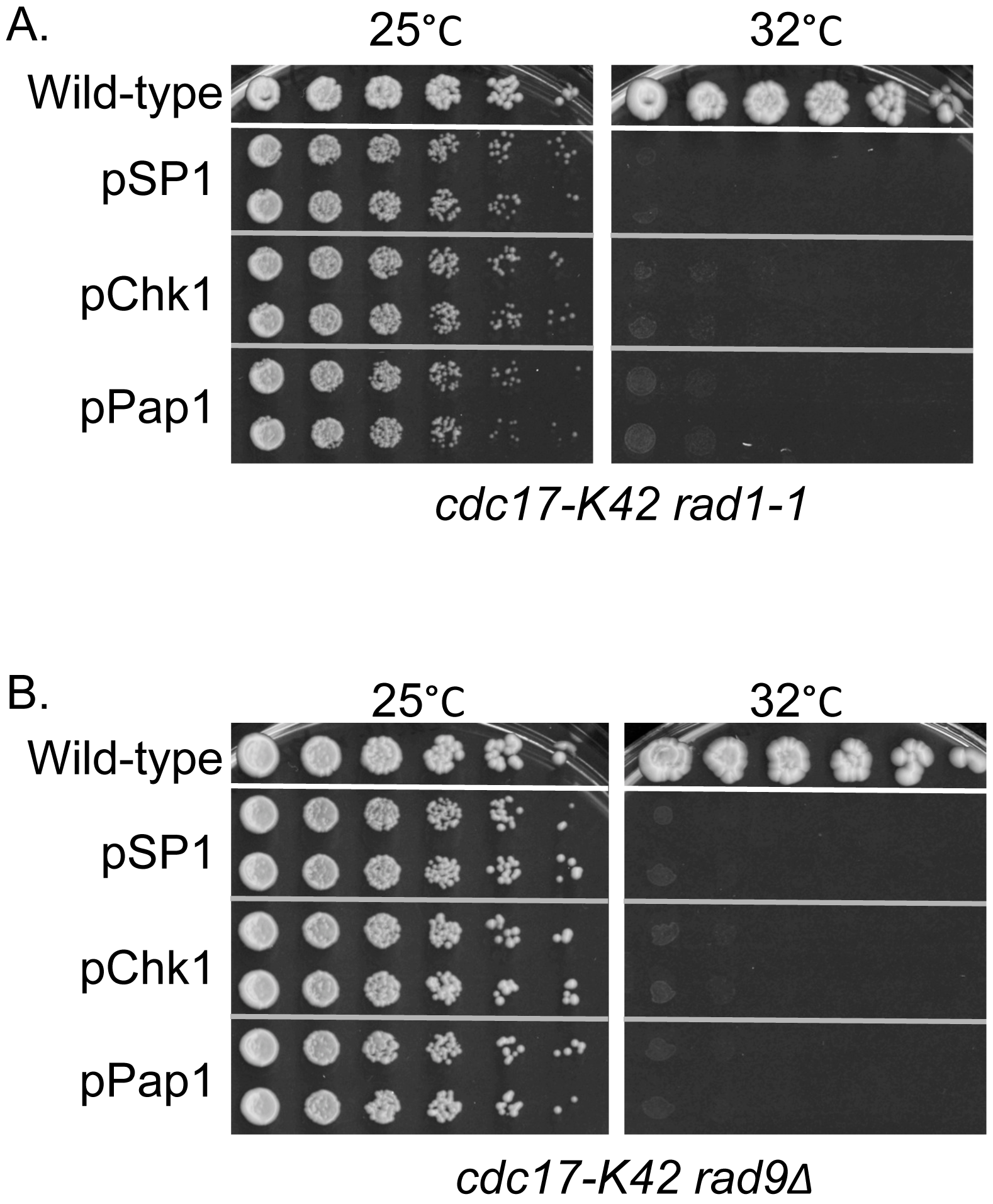

Supplement: Figure S2 — Pap1 is unable to rescue 9-1-1 complex mutants with compromised DNA ligase. Strains of the indicated genotypes were transformed with the indicated plasmids and grown to mid-log phase at 25°C. Serial dilutions were spotted at 25°C or restrictive temperature for the double mutants, as indicated, and incubated for 3 to 5 days. (A) cdc17-K42 rad1-1 (B) cdc17-K42 rad9Δ. (TIF) [file pone.0089936.s002.tif]

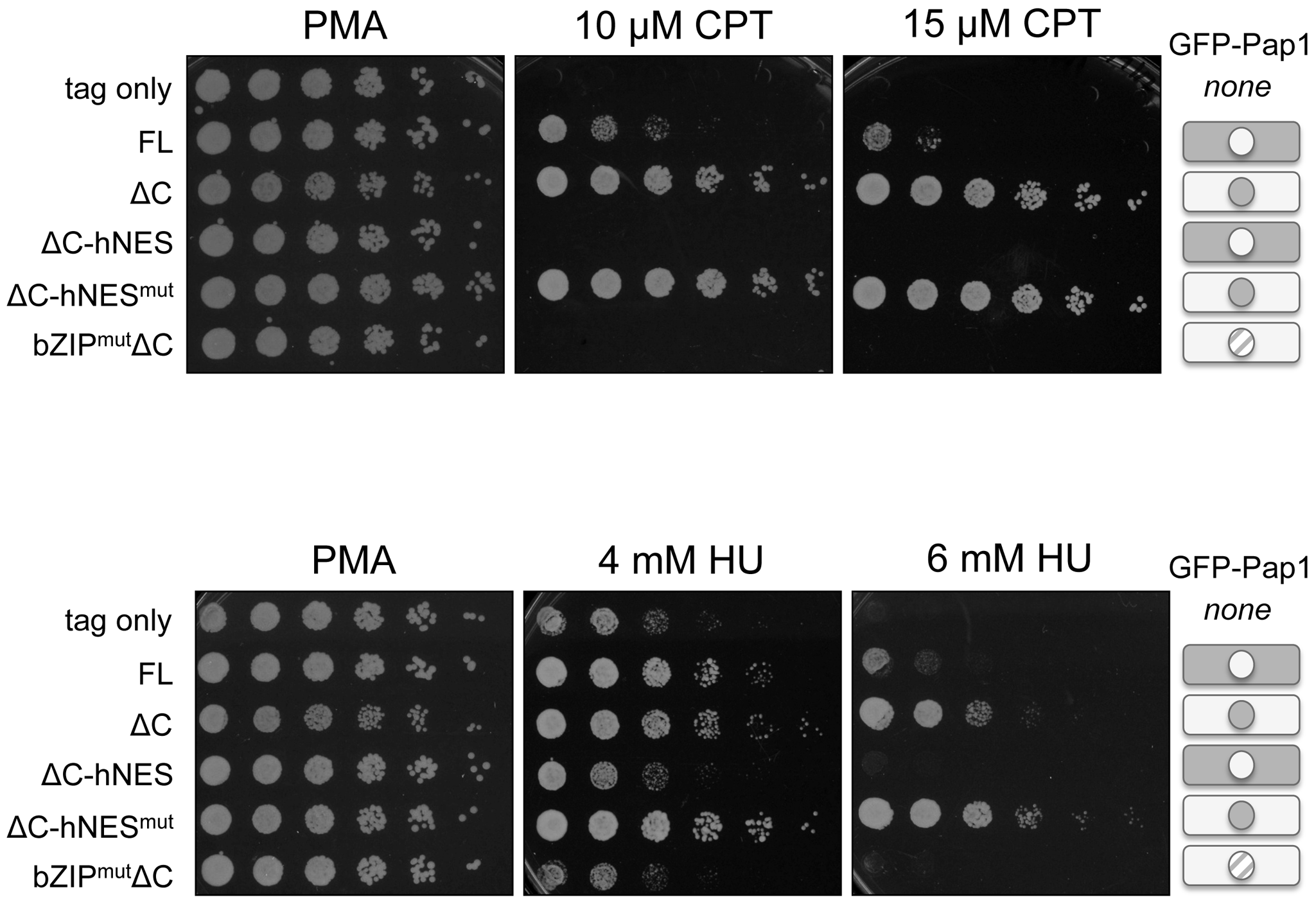

Supplement: Figure S3 — Pap1 that is nuclear and transcriptionally active confers resistance to cells deleted for chk1 when exposed to CPT or HU. (A) Cells of a chk1::ura4 strain with integrated alleles of the indicated Pap1 constructs expressed under control of the nmt41 promoter were assayed for survival in the presence of 10 or 15 µM CPT. (B) Strains as in A were assayed for survival in the presence of 4 or 6 mM HU. (TIF) [file pone.0089936.s003.tif]
