# Supplementary material for: The Oxidative Stress Responsive Transcription Factor Pap1 Confers DNA Damage Resistance on Checkpoint-Deficient Fission Yeast Cells
Source: PLoS One. 2014 Feb 25;9(2):e89936. doi: 10.1371/journal.pone.0089936 (PMC3934961; doi:10.1371/journal.pone.0089936)
Supplement: Table S1 — Yeast Strains Used. (DOCX) [file pone.0089936.s004.docx]

**Table S1. Yeast Strains Used**

| **Strains** | **Genotype** | **Source** |
| --- | --- | --- |
| SP6 | *h^-^ leu1-32* | Lab stock |
| NW158 | *h^+^ chk1::ura4 ade6-216 leu1-32 ura4-D18* | Lab stock |
| NW1509 | *h^+^ chk1D469G:HA ade6-216 leu1-32* | Lab stock |
| NW653 | *h^+^ cdc17-K42 chk1::ura4 ade6-216 leu1-32 ura4-D18* | Lab stock |
| AL106 | *cdc6-23 chk1::ura4 leu1-32 ura4-D18* | Yin et al., 2008 |
| DP55 | *cdc21-M68 chk1::ura4 leu1-32 ura4-D18* | Yin et al., 2008 |
| DP51 | *cdc18-K46 chk1::ura4 leu1-32 ura4-D18* | Yin et al., 2008 |
| NW116 | *h^-^ wee1-50 chk1::ura4 leu1-32 ura4-D18* | Lab stock |
| NW2859 | *rad3-T80 chk1D469G:HA ade6-210 leu1-32* | This study |
| NW215 | *h^+^ rad1::ura4 ura4-D18 leu1-32* | Lab stock |
| NW241 | *h^-^ rad9::ura4 ura4-D18 leu1-32* | Lab stock |
| NW2860 | *sty1::ura4 chk1D469G:HA ade6-216 leu1-32 ura4-D18* | This study |
| NW2861 | *srx1::KanMX6 chk1D469G:HA ade6-216 leu1-32 [*derived from *EA38 (h- leu1 srx1::KanMX6), gift of Dr. Elena Hidalgo]* | This study |
| SS767 | *nmt41::GFP-Pap1(leu1^+^) ura4-D18 ade6-210* | Fleig et al., 2000 |
| NW2863 | *chk1::ura4 nmt1::GFP-Pap1(leu1^+^) ura4-D18 ade6-210 ura4-D18* | This study |
| NW2864 | *chk1D469G:HA nmt1::GFP-Pap1(leu1^+^) ura4-D18 ade6-216* | This study |
| KSP2133 | *h^-^ pap1::ura4 ura4 leu1^+^::[Pnmt41-6HisFlagGFP]* | Kitamura, et al. 2011 |
| KSP2134 | *h^-^ pap1::ura4 ura4 leu1^+^::[Pnmt41-6HisFlagGFP-pap1ΔC]* | Kitamura, et al. 2011 |
| KSP2137 | *h^-^ pap1::ura4 ura4 leu1^+^::[Pnmt41-6HisFlagGFP-pap1ΔC-NES^PK1^]* | Kitamura, et al. 2011 |
| KSP2139 | *h^-^ pap1::ura4 ura4 leu1^+^::[Pnmt41-6HisFlagGFP-pap1ΔC-NES^PK1^.mut^P11^]* | Kitamura, et al. 2011 |
| KSP2264 | *h^-^ pap1::ura4 ura4 leu1^+^::[Pnmt41-6HisFlagGFP-pap1-bZIP^GAA^ΔC]* | Kitamura, et al. 2011 |
| KSP2783 | *h^-^ pap1::ura4 ura4 leu1^+^::[Pnmt41-6HisFlagGFP-pap1-FL]* | Kitamura, et al. 2011 |
| NW2871 | *chk1::arg3 pap1::ura4 arg3-D4 ura4 leu1^+^::[Pnmt41-6HisFlagGFP]* | This study |
| NW2872 | *chk1::arg3 pap1::ura4 arg3-D4 ura4 leu1^+^::[Pnmt41-6HisFlagGFP-pap1ΔC]* | This study |
| NW2873 | *chk1::arg3 pap1::ura4 arg3-D4 ura4 leu1^+^::[Pnmt41-6HisFlagGFP-pap1ΔC-NES^PK1^]* | This study |
| NW2874 | *chk1::arg3 pap1::ura4 arg3-D4 ura4 leu1^+^::[Pnmt41-6HisFlagGFP-pap1ΔC-NES^PK1^.mut^P11^]* | This study |
| NW2875 | *chk1::arg3 pap1::ura4 arg3-D4 ura4 leu1^+^::[Pnmt41-6HisFlagGFP-pap1-bZIP^GAA^ΔC]* | This study |
| NW2876 | *chk1::arg3 pap1::ura4 arg3-D4 ura4 leu1^+^::[Pnmt41-6HisFlagGFP-pap1-FL]* | This study |
| NW2877 | *chk1D469G:HA pap1::ura4 ura4 leu1^+^::[Pnmt41-6HisFlagGFP] ade6-216* | This study |
| NW2878 | *chk1D469G:HA pap1::ura4 ura4 leu1^+^::[Pnmt41-6HisFlagGFP-pap1ΔC] ade6-216* | This study |
| NW2879 | *chk1D469G:HA pap1::ura4 ura4 leu1^+^::[Pnmt41-6HisFlagGFP-pap1ΔC-NES^PK1^] ade6-216* | This study |
| NW2880 | *chk1D469G:HA pap1::ura4 ura4 leu1^+^::[Pnmt41-6HisFlagGFP-pap1ΔC-NES^PK1^.mut^P11^] ade6-216* | This study |
| NW2881 | *chk1D469G:HA pap1::ura4 ura4 leu1^+^::[Pnmt41-6HisFlagGFP-pap1-bZIP^GAA^ΔC] ade6-216* | This study |
| NW2882 | *chk1D469G:HA pap1::ura4 ura4 leu1^+^::[Pnmt41-6HisFlagGFP-pap1-FL] ade6-216* | This study |
| NW2883 | *cdc17-K42 chk1D469G:HA pap1::ura4 ura4 leu1^+^::[Pnmt41-6HisFlagGFP] ade6-216* | This study |
| NW2884 | *cdc17-K42 chk1D469G:HA pap1::ura4 ura4 leu1^+^::[Pnmt41-6HisFlagGFP-pap1ΔC] ade6-216* | This study |
| NW2885 | *cdc17-K42 chk1D469G:HA pap1::ura4 ura4 leu1^+^::[Pnmt41-6HisFlagGFP-pap1ΔC-NES^PK1^] ade6-216* | This study |
| NW2886 | *cdc17-K42 chk1D469G:HA pap1::ura4 ura4 leu1^+^::[Pnmt41-6HisFlagGFP-pap1ΔC-NES^PK1^.mut^P11^] ade6-216* | This study |
| NW2887 | *cdc17-K42 chk1D469G:HA pap1::ura4 ura4 leu1^+^::[Pnmt41-6HisFlagGFP-pap1-bZIP^GAA^ΔC] ade6-216* | This study |
| NW2888 | *cdc17-K42 chk1D469G:HA pap1::ura4 ura4 leu1^+^::[Pnmt41-6HisFlagGFP-pap1-FL] ade6-216* | This study |
| NW2889 | *cdc6-23 chk1D469G:HA pap1::ura4 ura4 leu1^+^::[Pnmt41-6HisFlagGFP] ade6-216* | This study |
| NW2890 | *cdc6-23 chk1D469G:HA pap1::ura4 ura4 leu1^+^::[Pnmt41-6HisFlagGFP-pap1ΔC] ade6-216* | This study |
| NW2891 | *cdc6-23 chk1D469G:HA pap1::ura4 ura4 leu1^+^::[Pnmt41-6HisFlagGFP-pap1ΔC-NES^PK1^] ade6-216* | This study |
| NW2892 | *cdc6-23 chk1D469G:HA pap1::ura4 ura4 leu1^+^::[Pnmt41-6HisFlagGFP-pap1ΔC-NES^PK1^.mut^P11^] ade6-216* | This study |
| NW2893 | *cdc6-23 chk1D469G:HA pap1::ura4 ura4 leu1^+^::[Pnmt41-6HisFlagGFP-pap1-bZIP^GAA^ΔC] ade6-216* | This study |
| NW2894 | *cdc6-23 chk1D469G:HA pap1::ura4 ura4 leu1^+^::[Pnmt41-6HisFlagGFP-pap1-FL] ade6-216* | This study |
| NW2895 | *cdc17-K42 rad1-1 leu1-32 ura4-D18* | This study |
| NW2896 | *cdc17-K42 rad9::ura4 leu1-32 ura4-D18 ade6-216* | This study |
| NW2897 | *cdc6-23 rad1-1 leu1-32 ade6-216* | This study |
